# Urinary 8-Hydroxy-2′-deoxyguanosine (8-OHdG) Concentrations and Menstrual Cycle Characteristics in Female University Students

**DOI:** 10.3390/ijerph15122616

**Published:** 2018-11-22

**Authors:** Shoko Konishi, Jun Yoshinaga, Yukiko Nishihama, Yu Onoda, Youichi Chisaki, Hideki Imai

**Affiliations:** 1 Department of Human Ecology, School of International Health, Graduate School of Medicine, The University of Tokyo, 7-3-1 Hongo, Bunkyo-ku, Tokyo 113-0033, Japan; 2 Department of Anthropology, University of Washington Box 353100 Seattle, Washington 98195, DC, USA; 3 Faculty of Life Sciences, Toyo University, 1-1-1 Izumino, Itakura, Ora, Gunma 374-0113, Japan; yoshinaga@toyo.jp; 4 National Institute for Environmental Studies, 16-2 Onogawa, Tsukuba 305-8506, Japan; nishihama.yukiko@nies.go.jp; 5 Department of Environment Systems, Graduate School of Frontier Science, The University of Tokyo, 5-1-5 Kashiwanoha, Kashiwa, Chiba 277-0882, Japan; 6 Institute of Environmental Ecology, IDEA Consultants, Inc., 1334-5 Riemon, Yaizu, Shizuoka 421-0212, Japan; ond20318@ideacon.co.jp (Y.O.); csk17405@ideacon.co.jp (Y.C.); 7 Faculty of Nursing, Tokyo Healthcare University, 2-5-1 Higashigaoka, Meguro, Tokyo 152-8558, Japan; h-imai@thcu.ac.jp

**Keywords:** menstrual cycle regularity, menstrual pain, oxidative stress, young women

## Abstract

Higher concentrations of oxidative stress biomarkers are found in women with polycystic ovary syndrome (PCOS) and endometriosis, conditions linked to irregular menstrual cycles and menstrual pain. The aim of the present study was to test whether women with higher oxidative stress are more likely to show irregular menstrual cycles and severe menstrual pain compared with women with lower oxidative stress. A cross-sectional study was conducted targeting female university students with a mean (SD) age of 20.5 (1.8) years (*n* = 188). Participants completed a questionnaire on reproductive characteristics and anthropometry and kept a menstrual cycle diary for 5 consecutive months. Urinary 8-hydroxy-2′-deoxyguanosine (8-OHdG), cotinine, and creatinine concentrations were measured once during the study period. The mean (SD) value of the urinary 8-OHdG concentration was 4.7 (2.0) μg/g of creatinine. A total of 1021 menstrual cycles were recorded. The participants were categorized as either having regular (68%) or irregular (18%) cycles or oligomenorrhea (13%) or polymenorrhea (1%). The urinary 8-OHdG concentration did not significantly differ across menstrual cycle regularity or pain categories. Even after adjusting for age, body mass index (BMI), and urinary cotinine concentrations, having irregular cycles or more severe menstrual pain was not associated with urinary 8-OHdG concentration.

## 1. Introduction

Menstrual cycle length is highly variable across and within women. Sources of variability include age [1], obesity [2,3], smoking [2], alcohol [4] and caffeine intake [5], and physical activity [4]. Previously, we reported that none of the examined lifestyle factors, i.e., alcohol and caffeine intake or physical activity, could explain the variability in menstrual cycles in a sample of Japanese women [6]. Using the data obtained from the same project [6], the present study aimed to test whether urinary measures of oxidative stress is associated with the variation in menstrual cycle characteristics, i.e., cycle regularity and menstrual pain.

We hypothesized that compared to women with a lower oxidative stress, women with a higher oxidative stress are more likely to have irregular menstrual cycles and more severe menstrual pain. Higher concentrations of oxidative stress biomarkers are found in women with polycystic ovary syndrome (PCOS) [7] and endometriosis [8], although urinary 8-OHdG was not included as an oxidative stress biomarker in the systematic review [7]. An irregular menstrual cycle is one of the major clinical symptoms of PCOS [9], and severe menstrual pain has been reported in endometriosis patients [10,11]. Previous systematic reviews suggest that oxidative stress plays an important role in the pathological process of both endometriosis [8] and PCOS [7]. Considering that oxidative stress is linked to reproductive disorders that are associated with irregular and more painful menstrual cycles, we hypothesized that higher oxidative stress is associated with irregular cycles and more severe menstrual pain.

To characterize menstrual cycle regularity, we conducted a 5-month prospective cohort study via the self-recording of menstrual bleeds. With the prospective recording, we could accurately categorize women with regular or irregular cycles. As a biomarker of oxidative stress, we used the urinary concentration of 8-hydroxy-2’-deoxyguanosine (8-OHdG). This urinary biomarker was selected because it is noninvasive, which could reduce the burden on study participants. The urinary concentration of 8-OHdG reflects the average rate of oxidative damage in the total body, rather than the oxidative stress levels in any specific organs or tissues [12]. This specific biomarker reflects the balance between oxidative damage and repair rate [12]. Using the prospectively recorded menstrual bleed data, we tested the following hypothesis: women with higher oxidative stress are more likely to have irregular cycles and severe menstrual pain, and women with lower oxidative stress are more likely to have regular cycles and less severe menstrual pain.

## 2. Materials and Methods

### 2.1. Subjects and Protocol

The data were obtained as a part of a research project to examine the effect of exposure to environmental chemicals on reproductive performance since 2012 [6,13,14]. The study was conducted in accordance with the Declaration of Helsinki. Institutional review board (IRB) approval was obtained from Tokyo Healthcare University and from the Graduate School of Frontier Sciences, The University of Tokyo (12-1-010 for Tokyo Healthcare University; 12-36 for the Graduate School of Frontier Sciences, The University of Tokyo). From 2012 to 2014, a total of 422 students were given an explanation about the objectives and procedure of the study and asked to participate. A total of 345 female students signed the informed consent and agreed to participate.

This paper is based on a cross-sectional study that included a one-time urine collection, a questionnaire survey, and a menstrual-cycle diary recorded prospectively for 5 consecutive months. One-time urine collection was conducted during the diary-recording period, irrespective of menstrual cycle phase, because urinary concentrations of 8-OHdG do not significantly differ across a menstrual cycle [15]. The questionnaire included questions on age, height, and weight. At enrollment, participants received a sheet to record their menstrual bleed episode for 5 consecutive months. At the end of the diary-keeping period, the women were asked about menstrual pain and oral contraceptive use during the diary-keeping period. Use of oral contraceptives before the present study was not asked and thus was not considered in the analysis. Participants who used oral contraceptive during the diary-keeping period (11 out of 199 women who provided the complete dataset) were excluded from the analytic sample. Each woman was asked to describe the most severe menstrual pain episode during the 5-month diary-keeping period using four categories: very mild (“almost none”); mild (“felt pain but had no problem in daily activities”); moderate (“could go out, but felt difficulty in doing physical exercise”); or severe (“too painful to get up from a bed”). 

### 2.2. Laboratory Analyses

Urine samples were stored at −80 °C until the assays were performed. Urinary concentrations of cotinine and 8-OHdG were measured at IDEA Consultants, Inc., Shizuoka, Japan, where simultaneous determination of the two components by column-switching LC-MS/MS was employed [16]. The accuracy of cotinine analysis was extensively validated through the analyses of urine-based NIST SRM 3673 certified reference materials, which were organic contaminants in nonsmokers’ urine (frozen). The accuracy of the 8-OHdG analysis was judged to be reliable with a high recovery rate of 95.4% [16]. Urinary creatinine concentrations were measured using a commercial kit based on the Jaffe reaction (LabAssayTM, FUJIFILM Wako Pure Chemical, Osaka, Japan). Urinary 8-OHdG and cotinine concentrations were divided by the creatinine concentration to adjust for hydration status. 

### 2.3. Definition of Menstrual Cycle Regularity

The mean cycle length was first calculated for each woman using noncensored cycles. If any censored cycle was longer than the calculated mean cycle length of the same woman, the cycle length of the censored cycle was also used to calculate the mean cycle length of that woman [6]. Distribution of cycle lengths separately for censored and noncensored cycles were described in histograms.

Characteristics of menstrual cycle was defined following the methodology described elsewhere [13,17] with some modifications. We categorized each woman into one of four categories of menstrual-cycle regularity: regular cycles with a mean cycle length between 22 and 41 days; irregular cycles with a mean cycle length between 22 and 41 days and one or more cycles <22 (excluding censored cycles) or >41 days (both censored and noncensored); oligomenorrhea with a mean cycle length of >41 days; or polymenorrhea with a mean cycle length of <22 days. Urinary 8-OHdG concentrations adjusted for creatinine were compared across the four groups of menstrual cycle regularity, and across the four menstrual pain categories using boxplots.

### 2.4. Statistical Analyses

Menstrual cycle regularity was further categorized into two groups: regular and irregular (including irregular, oligomenorrhea, and polymenorrhea). Similarly, menstrual pain was dichotomized: milder (combining “very mild” and “mild”) or more severe (“combining “moderate” and “severe”). A logistic regression analysis was used to test whether an irregular menstrual cycle or severe menstrual pain was associated with higher 8-OHdG concentrations while adjusting for age, body mass index (BMI), and urinary cotinine concentrations. Odds ratios and 95% confidence intervals (CI) of having irregular cycles were estimated with the reference category of regular cycles (odds ratio = 1.0). Similarly, odds ratios and 95% CI of having severe menstrual pain was estimated with the reference category of milder menstrual pain (odds ratio = 1.0). Statistical analyses were performed with R version 3.4.0 [18].

## 3. Results

**Figure 1:**
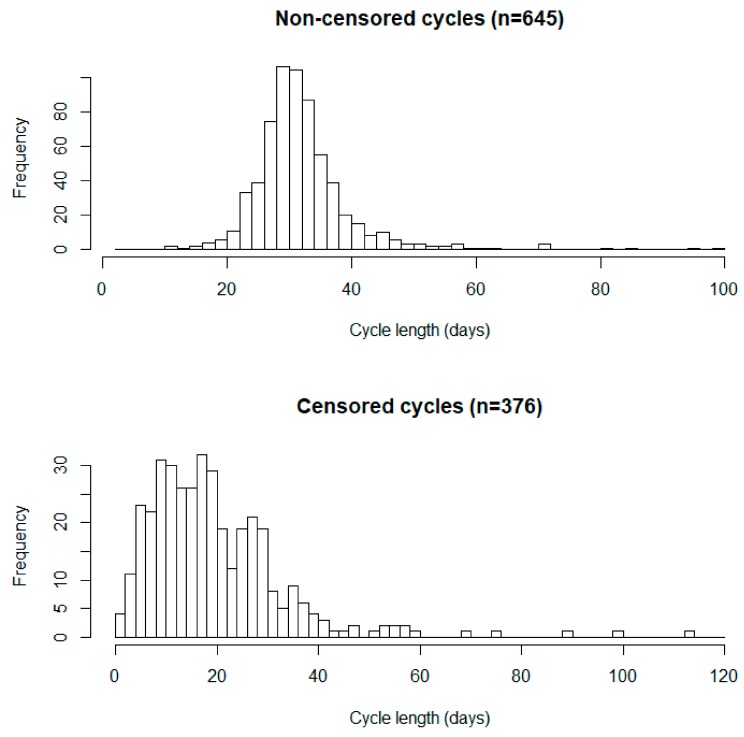
Distribution of cycle length for *n* = 645 noncensored and *n* = 376 censored cycles.

**Figure 2:**
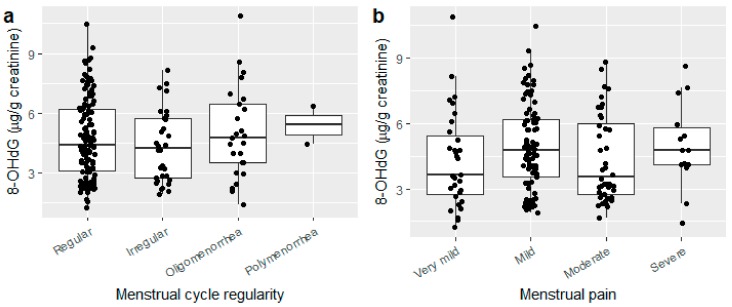
Association between urinary 8-OHdG concentration and menstrual cycle characteristics: (**a**) regularity and (**b**) menstrual pain (*n* = 182).

Among the 345 women who enrolled, 188 provided a complete data set and used no oral contraceptives during the 5-month diary-keeping period. The mean (SD) age was 20.5 (1.8) years, the mean (SD) BMI was 20.9 (2.2) kg/m^2^, and the median (interquartile range) urinary 8-OHdG concentration was 5.1 (3.1–8.3) ng/mL (Table 1). A total of 1021 menstrual cycles were recorded. The distribution of cycle length of noncensored (*n* = 645) and censored (*n* = 376) cycles are shown in Figure 1. Urinary 8-OHdG concentration did not differ significantly across menstrual cycle regularity or pain categories (Figure 2a,b). Having irregular cycles or more severe menstrual pain was not associated with urinary 8-OHdG concentration (Table 2).

## 4. Discussion

We found no significant association between oxidative stress as measured by urinary 8-OHdG concentration and menstrual cycle characteristics, i.e., cycle regularity and menstrual pain. The mean (SD) urinary 8-OHdG concentration in the present sample, i.e., 4.7 (2.0) μg/g of creatinine, was similar to the reported values in previous studies (i.e., a mean (SD) of 5.1 (3.0) μg/g of creatinine in healthy subjects [19], 3.4 (1.2) μg/g of creatinine in filling station attendants [20], 4.6 (1.6) μg/g of creatinine in female nonsmokers, 4.2 (1.3) μg/g of creatinine in female passive smokers, and 5.1 (0.8) μg/g of creatinine in female smokers [21]). These previous values were measured using HPLC. The present analytical methods were extensively validated for accuracy [16]; thus, the analytical values were considered reliable.

The lack of association between the urinary 8-OHdG level and menstrual cycle characteristics observed in the present study may suggest that cellular oxidative stress is not associated with menstrual pain or cycle irregularity. We assumed that severe menstrual pain and irregular menstrual cycles were linked to reproductive disorders such as PCOS and endometriosis, but it may be the case that physiological variability unrelated to these disorders plays an important role in the variability of female menstrual cycles. In the present study, we could only examine associations and thus underlying physiological mechanisms of the non-association should be further investigated in future studies. Another possible reason for the non-association is that this specific biomarker reflects the total oxidative stress level of a whole body [21], rather than oxidative stress level in specific organs. In other words, urinary 8-OHdG may not be able to capture the oxidative stress level in reproductive organs, e.g., ovaries and uterus, which may be associated with menstrual cycle regularity and menstrual pain. Assuming that a large increase in oxidative stress is required to alter the menstrual cycle or induce severe menstrual pain, a positive association between 8-OHdG and menstrual cycle characteristics should be observed. However, this association was not observed in this study. Therefore, we suggest that the lack of specificity of the urinary 8-OHdG concentration may be one of the reasons why we did not observe a significant association between the biomarker and the menstrual cycle characteristics. In addition, urinary 8-OHdG concentrations vary within individuals [21]; thus, we could not quantify the oxidative stress level for a longer time scale. A further study with other biomarkers of oxidative stress, possibly with multiple sampling, is warranted to examine the association between oxidative stress and menstrual cycle characteristics.

Irregular menstrual cycles and severe menstrual pain have been reported in patients with PCOS [9] or endometriosis [10,11]. A number of case-control studies, but not all, have reported significantly higher concentrations of oxidative stress biomarkers among PCOS [7] and endometriosis [8] patients compared with healthy controls. Based on these previous studies, we hypothesized that women with higher oxidative stress are more likely to have physiological traits similar to PCOS and endometriosis patients and thus exhibit irregular cycles and more severe menstrual pain. However, we found no significant association between urinary 8-OHdG concentration and cycle regularity or menstrual pain. One possibility is that concentrations of urinary 8-OHdG, a biomarker reflecting the oxidative stress level of the entire body [21], are elevated as a result of diseases such as PCOS and endometriosis. Thus, a subtle increase in urinary 8-OHdG concentration is not directly linked to these reproductive disorders. It is also possible that urinary 8-OHdG concentration is not associated with PCOS or endometriosis; only other biomarkers of oxidative stress are linked to them. Biomarkers that have showed significantly higher levels in PCOS patients compared with controls include malondialdehyde, asymmetric dimethylarginine, and superoxide dismutase activity, but not 8-OHdG, as described in a prior review paper [7]. In another review paper on endometriosis and oxidative stress [8], 36 oxidative stress biomarkers were listed, among which 23 biomarkers were significantly higher in endometriosis patients compared with controls. Concentrations of 8-OHdG in ovarian cysts or tissue were significantly higher in endometriosis patients compared to controls [22,23,24], whereas another cited study reported no significant differences in 8-OHdG in tissue between patients and controls [25]. 

Variability of menstrual cycle can be attributed to factors other than PCOS or endometriosis. Younger and older reproductive age is associated with more irregular cycles [1]; however, age cannot explain the regularity of cycles in this study because 90% of our samples were from women between 19 and 21 years of age. Lifestyle factors, such as BMI, age at menarche, alcohol and caffeine intake, and physical activity, were not associated with menstrual cycle length or variability in the sample of women taken from the same project as the present paper [6]. Considering that a shorter cycle length was found in women with higher urinary levels of total paraben and butyl paraben [14], it is possible that exposure to paraben and other environmental chemicals may play a role in menstrual cycle variability. A further study is needed to investigate the sources of variability in menstrual cycle characteristics and whether the variability of cycles at younger ages is associated with fecundability of women at later stages of life.

In addition to the limitation of the biomarker employed, the cross-sectional design with a single urine collection is also a limitation. The strength of this study was due to prospectively collected menstrual cycle data with a relatively large sample size. Self-reported cycle length, commonly used in previous studies, has been reported to be less accurate for women with irregular cycles [26].

## 5. Conclusions

No association between urinary concentration of 8-OHdG and menstrual cycle characteristics was observed.

## Figures and Tables

**Table 1:** Basic characteristics of the participants (*n* = 188).

Variables	Mean (SD), Median (Interquartile Range), and Sample Size (%)
Age (y) a	20.5 (1.8)
BMI (kg/m2) b	20.9 (2.2)
Cycle length (day)	34.4 (26.7–35.8)
Cycle regularity c	
Regular	*n* = 127 (68%)
Irregular	*n* = 34 (18%)
Oligomenorrhoea	*n* = 25 (13%)
Polymenorrhea	*n* = 2 (1%)
Menstrual pain	
Very mild	*n* = 31 (16%)
Mild	*n* = 95 (51%)
Moderate	*n* = 45 (24%)
Severe	*n* = 16 (9%)
N/A	*n* = 1 (1%)
Urinary 8-OHdG (ng/mL)	5.1 (3.1–8.3)
Urinary 8-OHdG, creatinine adjusted (μg/g creatinine)	4.5 (3.1–6.1)
Urinary cotinine (ng/mL)	0.7 (0.2–2.4)
Urinary cotinine (μg/g creatinine)	0.7 (0.2–2.1)

^a^*n* = 10 did not report ages. ^b^ *n* = 29 reported no BMI values. ^c^ Categorization was based on Reference [17] with some modifications: regular cycles with a mean cycle length between 22 and 41 days; irregular cycles with a mean cycle length between 22 and 41 days and one cycle of <22 (excluding censored cycles) or >41 days (both censored and noncensored); oligomenorrhea with a mean cycle length of >41 days or polymenorrhea with a mean cycle length of <22 days.

**Table 2:** Odds ratios and 95% CI for having irregular cycles and menstrual pain.

	OR (95% CI)	
	Irregular Cycle (*n* = 152)	Menstrual Pain (*n* = 151)
Age (y)	1.05 (0.87, 1.28)	1.13 (0.94, 1.43)
BMI (kg/m^2^)	0.97 (0.82, 1.13)	0.95 (0.80, 1.12)
8-OHdG (μg/g creatinine)	1.00 (0.84, 1.19)	0.90 (0.74, 1.08)
Cotinine (μg/g creatinine)	1.00 (0.99, 1.00)	1.00 (1.00, 1.01)
